# Lithotomy in the Female

**Published:** 1831

**Authors:** 


### Lithotomy in the Female.

In 1796, M. Larrey performed the operation of lithotomy on a female, æt. 34, at Toulon; she was cured on the 8th day, and that without incontinence of urine. M. Larrey avoided dividing the paries of the vagina. With a grooved staff and a bistoury, he cut to right and left between the parietes of the vagina and rami of the ischia. The stone was extracted without difficulty.

Here we may take an opportunity of reviewing a very useful work, which we have not previously done. It is a System of Operative Surgery, from the pen of Mr. Hargrave of Dublin, and we have no hesitation in saying, that it is greatly superior to the production of the late Mr. Averill. We shall at once do justice to the author for a long silence, give our readers a specimen of the style and matter of the volume, and put them in possession of the various methods of performing the operation of lithotomy in the female by the following quotation:—

"The methods by incising the bladder, to remove the calculus, are necessary to be adopted, when it is very voluminous; of these, five plans have been invented to permit the surgeon to accomplish his intentions.

The high operation. It is conducted on the same principle as in the male subject, and is less dangerous in the female than in the male, since there is very little probability of infiltration of urine succeeding to it, as it can be prevented by the introduction of a catheter into the bladder.

Lateral operation. The surgeon makes use of a straight and deeply-grooved staff, with any of the other cutting instruments that are required in the opposite sex. *First period.* The patient being secured in a similar manner as for the operation in the male, the labia are to be separated and the staff introduced into the bladder, and the presence of the stone verified; the groove of it is to be then lateralized, by turning it obliquely downwards, at the same time that it is raised to the pubic arch, which separates the urethra from the vagina. *Second period.* The operator then passes his knife along the groove, which is also held in the lateral position, dividing the urethra into the bladder, when a gush of urine flows. *Third period.* He then depresses his hand, withdraws the knife, and cuts obliquely downwards and outwards between the left ramus of the pubes and ischium, and the side of the vagina, so as to make a free opening. *Fourth period.* The finger is then passed into the bladder, on the blunt gorget, which allows the forceps to be carried along it to seize the stone for its extraction.

The advantages of this operation consist in affording a free passage for the extraction of the calculus, without producing the risk of a permanent incontinence of urine: but it is not unattended with some danger during its performance, from the following inadvertencies: the knife, if too much lateralized, may divide the pudic artery, while if the proper degree of lateralization is not given to it, the vagina will be laid open, and vesico-vaginal fistula be produced.

*The sub-pubic Operation, as performed by* M. Dubois. After the patient is placed in the proper position, *first period,* a straight grooved sound is introduced into the urethra, the groove being directed to the symphysis pubis; which is depressed with the left hand, to separate the canal from the pubic arch. *Second period.* The operator then conducts, by the groove, a scalpel, and divides the superior parietes of the urethra, with the neck of the bladder, of sufficient extent to allow the extraction of the calculus. *Third period.* The instruments being withdrawn, the finger is passed into the bladder to examine the size of the wound, and to guide the forceps into it. This operation may be performed with the bistoire caché, without any previous introduction of a staff.

As the soft parts in this situation are very lax and extensible, no impediment is offered to the withdrawal of the forceps charged with the stone: no vessels of any size are wounded by this plan, as none course in the line of the incisions.

*Sub-pubic Operation, as performed by* M. LISFRANC. *First period.* The patient being situated as in the other subpubic operations, and the labia divaricated, the operator introduces an ordinary staff into the urethra, and gives it in charge to an assistant, who presses it gently downwards, and with it the urethra and vagina; the surgeon then examines in the vagina with the index finger, for the course of the pudic arteries; after which, with a common bistoury, he makes a semilunar incision, beginning on a level with the right side of the urethra, and cuts parallel to the ascending ramus of the pubes, then to the symphysis, and terminates at a corresponding point on the opposite region of the urinary canal; the convexity of this incision looks upwards, and is distant about one line from the bones and symphysis pubis. The handle of the bistoury, when making this incision, should be a little lower than the point, which divides, cautiously, the different layers of tissues till the bladder is exposed. *Second period.* The bladder being exposed, the operator either may plunge his knife into it, and cut that viscus transversely, or he will find it more safe and expeditious, to introduce the thumb of the left hand into the vagina, and the index finger into the wound, when, by drawing gently the tissues held by them, the bladder is stretched, which can be opened either longitudinally or transversely. The longitudinal incision is parallel to the muscular fibres of the bladder, and is distant about sixteen lines, or an inch and a quarter at its superior extremity, from the peritoneum, while the transverse incision is perpendicular to these fibres, and is at a much greater distance from the peritoneum.

The inventor conceives that no danger is incurred of peritoneal inflammation, urinary fistulæ, or incontinence of urine, which too often succeed to the other sub-pubic methods.

*Observation.* I have occasionally performed this operation in the dead subject, and cannot coincide with M. Lisfranc as to its eligibility, either in the executive part or the extraction of the calculus; the first is effected in a slow and unsatisfactory manner, as the point of the knife can be alone employed to divide the parts; the incisions into the bladder are not guided by any defined course; hence the operator is liable to open the urethra at its point of junction with the bladder, or the canal may be actually cut across, even if the incisions are well directed; the bladder is opened in the narrowest part of the pelvic outlet, so that calculi of any size can scarcely pass through it without considerable dilatation of parts almost incapable of it: it is also necessary to press the neck of the bladder and urethra downward to the vagina in a very forcible manner, to afford space for the extraction of the forceps loaded with the calculus, which will cause not alone contusion, but often very serious laceration."

---

XLII.

